# Attentional control mediates the relationship between social anhedonia and social impairment

**DOI:** 10.3389/fpsyg.2014.01384

**Published:** 2014-12-03

**Authors:** Laura M. Tully, Sarah Hope Lincoln, Christine I. Hooker

**Affiliations:** Social Neuroscience and Psychopathology Laboratory, Department of Psychology, Harvard UniversityCambridge, MA, USA

**Keywords:** social anhedonia, attentional control, social functioning, psychosis-proneness, schizotypy, schizophrenia

## Abstract

Social anhedonia (SA), a trait-like disinterest in social contact and diminished capacity to experience pleasure from social interactions, is consistently associated with social impairments in both healthy and clinical populations. However, the mechanisms underlying the relationship between SA and social impairment are poorly understood. Attentional control, selecting and focusing on relevant information and inhibiting irrelevant, may be one such mechanism. We examined individual differences in SA, attentional control, and social impairment in 108 healthy adults. High SA related to low attentional control and high social impairment. Moreover, attentional control mediated the relationship between SA and social impairment, establishing attentional control as one mechanism underlying aberrations in the fundamental human need for social contact. Although both attentional deficits and social impairment have been separately noted in SA, the relationship between SA, attentional control and social impairment in this non-clinical sample reflects a novel contribution.

## INTRODUCTION

The desire for frequent and meaningful social interactions is a fundamental human motivation (Baumeister and Leary, 1995). Social anhedonia (SA), a trait-like disinterest in social contact and diminished capacity to experience pleasure from social interactions, is an example of when this need to belong goes awry (Silvia and Kwapil, 2011). Although socially anhedonic individuals report a genuine preference for solitude and reduced negative affect when alone (Brown et al., 2007; Kwapil et al., 2009), their asocial solitude negatively impacts their psychological well-being. High SA individuals report fewer social supports and less satisfaction with their existing social supports (Blanchard et al., 2011), avoidant attachment (Troisi et al., 2010), decreased social competence, and overall poor social functioning (Llerena et al., 2012) – all factors that are known to adversely impact important physical and mental health outcomes, possibly due to the lack of protective effects conveyed by social contact (Miller et al., 2008; Silvia and Kwapil, 2011). Indeed, high SA is consistently identified as a risk factor for psychiatric disorders (Watson and Naragon-Gainey, 2010), particularly schizophrenia-spectrum disorders. Meehl (1962) conceptualized SA as a feature of schizotypy – personality characteristics indicative of genetic liability for schizophrenia. Consistent with Meehl’s theory, SA is one of the strongest predictive traits of conversion to schizophrenia-spectrum disorders (Kwapil, 1998), and, similar to findings in non-clinical populations, is a key factor contributing to the characteristic social deficits in schizophrenia (Meehl, 1962; Blanchard et al., 1998, 2000). Collectively, existing evidence consistently associates high SA with poor social functioning in both healthy and clinical populations.

Although logically it follows that a reduced desire for social contact would lead to fewer friends and social engagements (i.e., poor social functioning), the underlying reason for *why* high SA individuals have poor social functioning is not entirely known. One proposal is that the consequences of SA may be related to deficits in attentional control, also termed effortful control, executive control, or cognitive control, and operationalized as the capacity to engage the inhibitory functions necessary to maintain task-relevant processing and goal oriented behavior (Derryberry and Reed, 2002; Lesh et al., 2011; Tully and Niendam, 2014). A core aspect of attentional control is the ability to inhibit prepotent responses in favor of subdominant ones – a self-regulatory mechanism that is likely a key component of successful social functioning (Heatherton and Wagner, 2011). Social interactions require the ability to filter out distracting/irrelevant information in order to attend to the relevant (e.g., in the “cocktail party” environment). This may be especially important in the context of emotional information: attentional control capabilities predict negative affect (Posner et al., 2002), response to conflict with a partner (Hooker et al., 2010), and response to social rejection (Gyurak et al., 2012), indicating that deficits in the ability to use attentional control to manage social/emotional information could harm social relationships over time, thereby negatively impacting social functioning. In this context, attentional control can be conceptualized as a domain-general system that assigns control resources to affect social behavior through multiple higher-level processes, such as the control of emotional information (Tully and Niendam, 2014), and other socially relevant processes such as theory of mind (ToM) and metacognition. This is consistent with the idea that neurocognitive impairments effect functioning via social cognitive impairments (Green et al., 2000; Schmidt et al., 2011), in which neurocognitive processes are the “building blocks” that precede socially relevant processes. In this study we examine attentional control as one of these neurocognitive processes.

Socially anhedonic but otherwise healthy individuals are impaired on tasks requiring attentional control, such as the Stroop paradigm (Giraldez et al., 2000) and the Wisconsin Card Sorting Task (Barrantes-Vidal et al., 2003). However, despite evidence demonstrating attentional control deficits in SA, the impact of these deficits on social functioning is rarely considered. In our previous work, we found high SA individuals demonstrated deficits in the attentional control of emotion information on an experimental task specifically designed to assess the ability to inhibit task-incongruent irrelevant negative faces, but these deficits did not relate to social impairments (Tully et al., 2012). However, highly specific experimental tasks may be too narrow to capture the effect of attentional control on social functioning. Here we sought to extend these findings by examining individual differences in attentional control as it naturally varies along a continuous dimension so as to better capture the multiple inhibitory demands of the social environment.

To test whether attentional control underlies the relationship between SA and social impairment, the present study investigates the relationship between individual differences in SA, attentional control, and social impairment in a large, representative, community sample. Specifically, we investigated whether attentional control mediates the relationship between SA and social impairment. Mediation analysis provides a meaningful statistical method for describing the mechanisms through which one variable exerts an effect on another (Hayes, 2009). We assessed SA using the Revised Social Anhedonia Scale (RSAS; Eckblad et al., 1982), attentional control using the Attentional Control Scale (ACS; Derryberry and Reed, 2002), and social impairment using the Social Adjustment Scale-Self-report (SAS-SR; Weissman et al., 1978). We hypothesized that: (1) high SA is associated with low attentional control and high social impairment, (2) low attentional control is associated with high social impairment, and (3) attentional control mediates the relationship between SA and social impairment.

## MATERIALS AND METHODS

### PARTICIPANTS AND PROCEDURES

One hundred and eight individuals participated in the study. Participants were recruited from the Greater Boston area via flyers posted in local cafes, community centers and on college campuses, as well as advertisements posted to online billboards (e.g., craigslist). Respondents to these flyers/advertisements contacted the lab and were scheduled for a screening appointment. Two hundred and twenty six individuals were screened for participation using the Structured Clinical Interview for DSM-IV Axis I Disorders (First et al., 2002), the Structured Clinical Interview for DSM-IV Personality Disorders (First et al., 1997), and the Weschler Abbreviated Scale of Intelligence (WASI; Wechsler, 1999). Inclusion criteria were: English as a first language, intelligence quotient (IQ) above 70, no history of head trauma, no neurological, or major medical illness, no current/past Axis I disorders, no current/past personality disorders (Axis II), no active substance abuse within the past 6 months, and no current/past substance dependence. Clinical interviews were conducted by two trained Ph.D. level clinical psychologists (Laura M. Tully, Sarah Hope Lincoln) and supervised by a licensed clinical psychologist (Christine I. Hooker). An independent clinician conducted reliability assessments on a random sample of ten clinical interviews, revealing a kappa of 0.67, indicative of substantial diagnostic agreement (Landis and Koch, 1977).

Of the 226 individuals screened for participation, 106 were excluded due to current/past Axis I disorders, three were excluded due to current/past personality disorders, and nine did not complete the study (missed appointments/withdrew following consent). Thus, a total of 108 participants met inclusion criteria for the study and completed the three study measures of SA, attentional control, and social impairment (detailed below). Demographics and sample characteristics of these 108 participants are presented in **Table 1**.

**Table 1:** Demographics and sample characteristics.

	Total sample	Male	Female	Gender differences
N	108	50	58	χ^2^(1) = 0.539, *p* = 0.441
Age	30.95 (12.87), [18-65]	32.32 (13.07), [18-64]	29.78 (12.69), [18-65]	*t*(106) = 1.024, *p* = 0.308
WASI IQ^a^	112.64 (12.92), [81-137]	112.63 (13.52), [81-137]	112.64 (12.51), [82-136]	*t*(105) = 0.002, *p* = 0.998
Years of education^b^	14.72 (2.11), [10-20]	14.27 (1.81), [10-18]	15.11 (2.27), [12-20]	*t*(104) = 2.08, *p* = 0.04
Level of education: *N* (%)^c^
10th grade	1 (0.9)	1 (2.0)	0 (0.0)	χ^2^(5) = 7.763, *p* = 0.170
High school	43 (39.8)	21 (42.0)	22 (37.9)	
In college	16 (14.8)	6 (12.0)	10 (17.2)	
Two year college	2 (1.9)	2 (4.0)	0 (0.0)	
Undergraduate degree	32 (29.6)	16 (32.0)	15 (25.9)	
Graduate degree	11 (10.2)	2 (4.0)	9 (15.5)	
Race: *N* (%)
White	77 (71.3)	35 (70.0)	42 (72.4)	χ^2^(3) = 1.330, *p* = 0.722
African American	17 (15.7)	10 (20.0)	7 (12.1)	
Asian American	8 (7.4)	3 (6.0)	5 (8.6)	
Multiracial	4 (3.7%)	2 (4.0)	2 (3.4)	
Latino/Hispanic: *N* (%)	4 (3.7%)	4 (8.0)	0 (0.0)	
Social anhedonia	12.71 (11.08), [0-40]	13.88 (11.22), [0-38]	11.71 (10.96), [0-40]	*t*(106) = 1.016, *p* = 0.312
Attentional control - total	56.44 (10.61), [25-78]	57.5 (11.02), [25-78]	55.53 (10.26), [33-74]	*t*(106) = 0.959, *p* = 0.340
Attentional focus	24.55 (5.44), [10-36]	25.5 (5.47), [10-36]	23.74 (5.32), [11-34]	*t*(106) = 1.690, *p* = 0.094
Attentional shifting	19.12 (3.34), [10-24]	19.24 (3.61), [10-24]	19.01 (3.13), [11-24]	*t*(106) = 0.343, *p* = 0.732
Divided attention	12.76 (3.48), [5-20]	12.76 (3.52), [5-20]	12.77 (3.47), [5-20]	*t*(106) = 0.023, *p* = 0.981
Social impairment^d^	58.85 (15.11), [36-109]	61.38 (16.90), [36-109]	56.72 (13.20), [36-89]	*t*(103) = 1.158, *p* = 0.116

*All data are presented as mean (SD), [range] unless otherwise noted; ^a^one participant did not complete the WASI IQ; ^b^three individuals did not report level of education; ^c^two individuals did not report race/ethnicity; ^d^ three participants did not complete the SAS-SR.*

Harvard University Institutional Review Board approved the study. Participants gave written informed consent and were paid for their participation.

### MEASURES

#### Social anhedonia

Participants completed the RSAS (Eckblad et al., 1982) – a 40 item true/false self-report scale measuring disinterest in social contact. Example items include: “Just being with friends can make me feel really good” (keyed false); “I attach very little importance to having close friends” (keyed true). Based on published norms, individuals are considered deviant on the RSAS if their score is greater than or equal to 1.96 standard deviation above the mean for their gender group: 16 or higher for females, 20 or higher for males (Kwapil, 1998). The RSAS has been extensively tested in a variety of clinical and non-clinical populations (Cohen et al., 2006; Kerns et al., 2008; Blanchard et al., 2011) and has been shown to have good psychometric properties (Leak, 1991; Blanchard et al., 2000; Fonseca-Pedrero et al., 2009; Chan et al., 2012). SA scores had high internal consistency in the current sample (α = 0.96).

#### Attentional control

Participants completed the ACS (Derryberry and Reed, 2002; Fajkowska and Derryberry, 2010), a 20-item questionnaire measuring three aspects of voluntary attention: focusing attention (nine items), shifting attention (six items), divided attention (five items). Example items: “I have a hard time concentrating when I’m excited about something” (focusing); “I can quickly shift from one task to another” (shifting); “My concentration is good even if there is music in the room around me” (divided). Items are rated on a 1-to-4 scale (1 = never; 4 = always). A full list of scale items are reported in **Table 2**. We used total ACS score in our primary analyses and conducted follow-up analyses using the three subscale scores.

**Table 2:** Attentional control scale subscale items and reliabilities.

Attentional control subscales	Attentional control scale questions. Rating scale: 1 = almost never; 4 = always	Coefficient alpha
Attentional focusing	When I need to concentrate and solve a problem, I have trouble focusing my attention. When I am working hard on something, I still get distracted by events around me. It’s very hard for me to concentrate on a difficult task when there are noises around. When I am reading or studying, I am easily distracted if there are people talking in the same room. When trying to focus my attention on something, I have difficulty blocking out distracting thoughts. I have a hard time concentrating when I’m excited about something. When concentrating I ignore feelings of hunger or thirst. After being interrupted or distracted, I can easily shift my attention back to what I was doing before. When a distracting thought comes to mind, it is easy for me to shift my attention away from it.	α = 0.83
Attentional shifting	I can quickly switch from one task to another. It takes me a while to get really involved in a new task. It is difficult for me to coordinate my attention between the listening and writing required when taking notes during lectures. I can become interested in a new topic very quickly when I need to. I have a hard time coming up with new ideas quickly. It is hard for me to break from one way of thinking about something and look at it from another point of view.	α = 0.76
Divided attention	My concentration is good even if there is music in the room around me. When concentrating, I can focus my attention so that I become unaware of what’s going on in the room around me. It is easy for me to read or write while I’m also talking on the phone. I have trouble carrying on two conversations at once. It is easy for me to alternate between two different tasks.	α = 0.73

Attentional control – total	-	α = 0.90

The ACS has high internal consistency; coefficient alpha for the ACS total score is high in this sample (α = 0.90), and comparable to prior literature (Fajkowska and Derryberry, 2010), as are the coefficients for each subscale (see **Table 2**). The ACS has also been shown to have good convergent and divergent validity with attentional and personality tests respectively (Fajkowska and Derryberry, 2010). Additionally, as part of a separate follow-up experiment a sub-sample of 54 participants in this study also completed the Digit Span Task – a well-established measure of working memory, which is reliant on attentional control mechanisms (Lesh et al., 2011). We conducted bivariate Pearson correlations examining the relationship between ACS scores and Digit Span performance in this sub-sample of 54 participants. Attentional control showed a moderately strong positive relationship with Digit Span such that higher Digit Span was associated with higher attentional control (*r* = 0.32, *p* = 0.02), specifically attentional focusing (*r* = 0.34, *p* = 0.012). This provides additional data that the ACS is a valid measure of attentional control mechanisms.

#### Social impairment

Participants completed the SAS-SR (Weissman et al., 1978), a selfreport questionnaire that consists of 54 questions assessing six major areas of functioning: work, social and leisure activities, relationships with extended family, role as marital partner, parental role, and role within the family unit. Areas of functioning are assessed across four categories: performance at expected tasks, level of conflict with people, interpersonal relations, and feelings and satisfactions. Area scores are averaged to create a single composite score of social impairment.

#### Intelligence

Full scale IQ scores were estimated using the matrix reasoning and vocabulary subtests of the WASI (Wechsler, 1999).

### STATISTICAL ANALYSIS

Data analysis was conducted with IBM SPSS 19.0. Chi square analysis and independent sample *t*-tests were used to assess gender differences and bivariate Pearson correlations were calculated to assess relationships between all variables. Due to three subjects missing data for one or more variables, the sample size used for the mediation analysis was 105.

#### Mediation analysis

We assessed mediation using bootstrapping, a non-parametric resampling procedure that constructs confidence intervals for the indirect effect of the proposed mediator (Hayes, 2009). Bootstrapping has several advantages over alternative methods. Unlike traditional approaches (e.g., Sobel’s *z*-test), bootstrapping does not assume a normal distribution of the indirect effect (MacKinnon et al., 2002), and simulation research indicates that it has more power and better control over type I error rates compared to the causal steps approach (Baron and Kenny, 1986) and product of coefficients approach (Sobel, 1982), particularly in small to moderate sample sizes (*N* < 500; Hoyle and Kenny, 1999; MacKinnon et al., 2002).

Here we assessed two mediation models: a single mediator model testing the effect of SA on social impairment through overall self-reported attentional control, followed by a multiple mediator model to determine the specific indirect effects of the three aspects of attentional control (focusing, divided, and shifting attention) on the relationship between SA and social impairment. We conducted bootstrap analysis with the SPSS macro INDIRECT from Preacher and Hayes (2008) to obtain estimates of the indirect effects and associated 95% confidence intervals using the recommended 5000 bootstrap samples. We used the SPSS macro RSQUARE from Fairchild et al. (2009) to calculate the portion of variance accounted for by the mediated effect of attentional control (*R*^2^_med_).

## RESULTS

**FIGURE 1:**
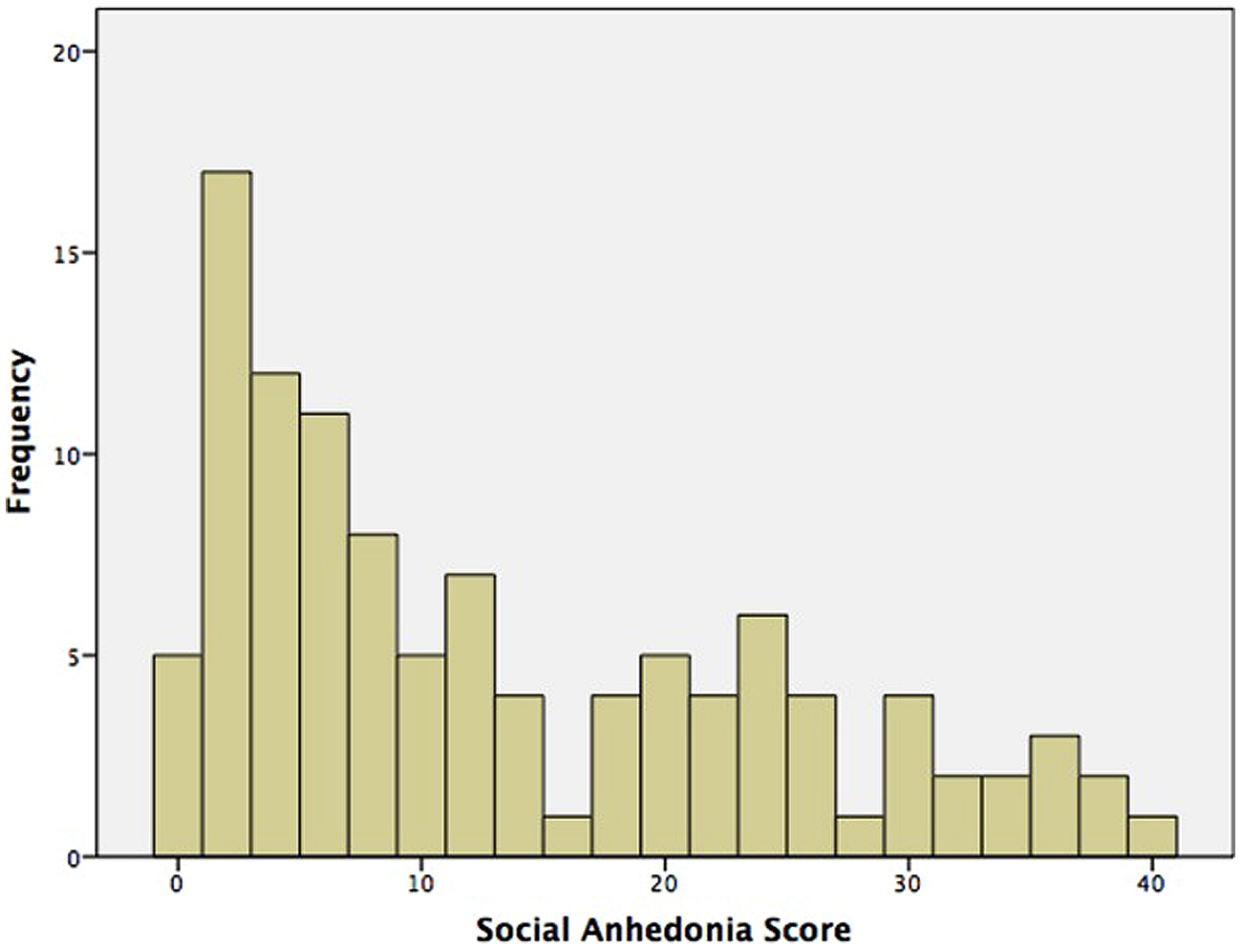
**Distribution of social anhedonia (SA) scores**.

**Table 1** presents demographics and sample characteristics. There were no gender differences on any demographic or self-report measures. SA spanned the full range of possible scores (0–40), and had acceptable spread (interquartile range = 19). 35 participants (32%) – 20 female (34% female participants) and 15 male (32% male participants) – were high deviant scorers per the cut offs reported by Kwapil (1998). The distribution of SA scores is presented in **Figure 1**.

**Table 3** presents bivariate Pearson correlation coefficients between IQ and all self-report variables. SA, attentional control, and social impairment were all significantly intercorrelated in the predicted directions; higher SA related to lower attentional control and greater social impairments, and lower attentional control related to higher social impairment. IQ did not significantly relate to any variables, indicating that these relationships are not due to IQ.

**Table 3:** Correlations between all variables.

	IQ	Social anhedonia	Social impairment
IQ	-	-	-
Social anhedonia	-0.11	-	-
Attentional control - total	0.06	-0.45**	-0.53**
Attentional focus	0.04	-0.38**	-0.54**
Attentional shifting	0.15	-0.57**	-0.54**
Divided attention	-0.04	-0.22*	-0.25*
Social impairment	-0.07	0.66**	-

p < 0.05 (2-tailed); **p < 0.001 (2-tailed).

### MEDIATION ANALYSIS

**FIGURE 2:**
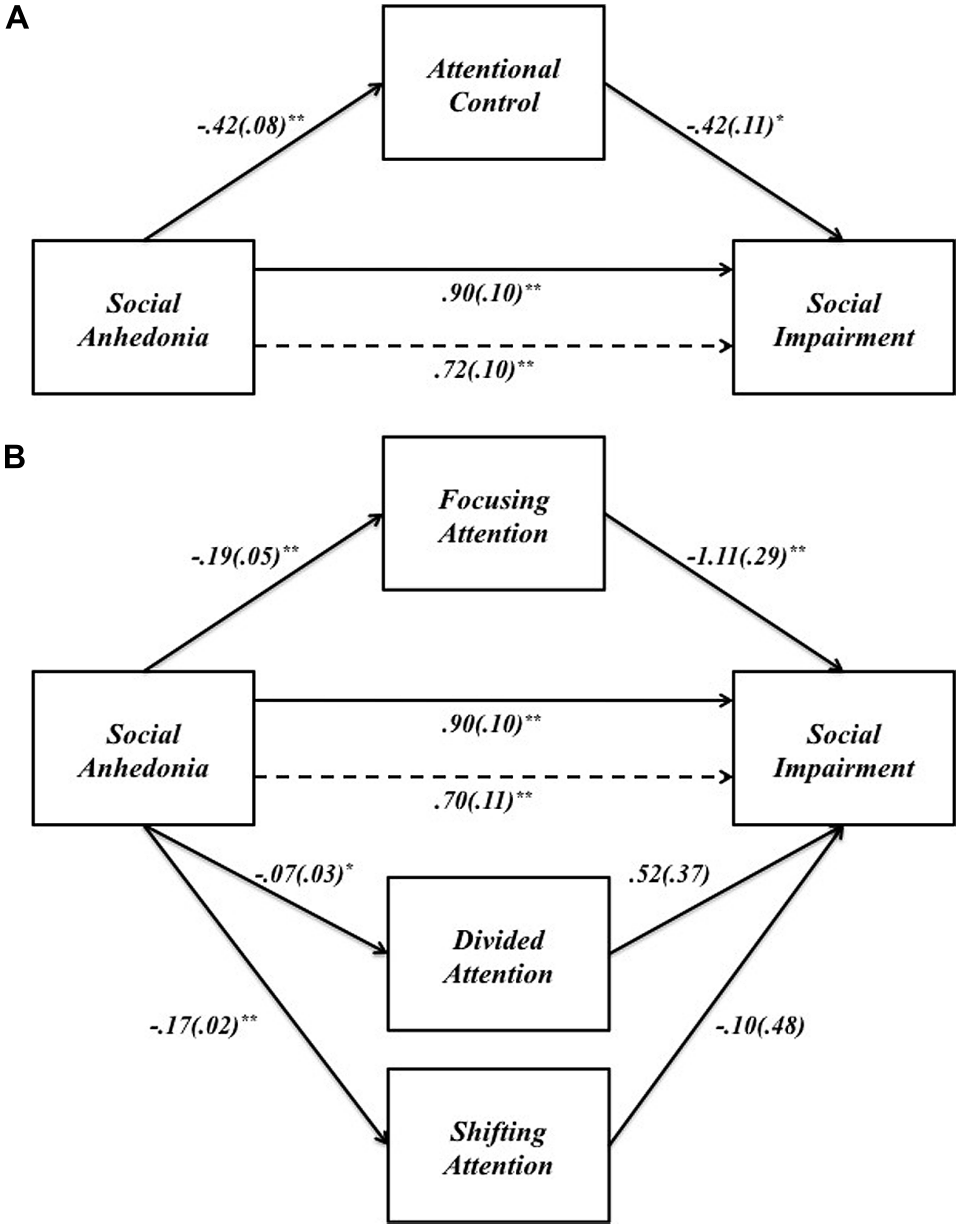
**(A)** The effect of SA on social impairment through attentional control. **(B)** The effect of SA on social impairment through the three components of attentional control: focusing, divided, and shifting attention. Unstandardized path coefficients (SE) shown for each path. *p < 0.05; **p < 0.001.

We assessed the single mediator model in which attentional control is hypothesized to mediate the relationship between SA and social impairment. All four paths were significant in the predicted directions (**Figure 2A**): SA had a total positive effect on social impairment (β = 0.90, *p* < 0.001), and a total negative effect on attentional control (β = -0.42, *p* < 0.001); attentional control had a direct negative effect on social impairment (β = -0.42, *p* = 0.001). Bootstrap analysis of the indirect effect (**Table 4A**) revealed a bias corrected 95% confidence interval excluding zero (CI_0.95_ = 0.08, 0.33), demonstrating that attentional control mediates the relationship between SA and social impairment. The direct effect of SA on social impairment, controlling for attentional control, remained significant (β = 0.72, *p* < 0.001), indicating that attentional control only partially mediates the relationship between SA and social impairment. The mediated effect of SA on social impairment through attentional control accounts for 19% of the variance in social impairment (*R*^2^_med_ = 0.19, CI_0.95_ = 0.08, 0.32).

**Table 4A:** Mediation of the effect of social anhedonia on social impairment through attentional control.

					BC 95% CI	
Indirect effect	Coefficient	Point estimate	Bias	SE	Lower	Upper
Indirect effect of SA on social impairment through attentional control	0.177	0.178	0.002	0.061	0.079	0.327

We examined the specific indirect effects of the three components of attentional control in a multiple mediator model (**Figure 2B**). SA had negative effects on all three components of attentional control: focusing (β = -0.19, *p* < 0.001), divided (β = -0.07, *p* < 0.05), and shifting attention (β = -0.17, *p* < 0.001). However, only focusing attention had a significant indirect effect on social impairment (β = -1.11, *p* < 0.001); all other paths between components of attentional control and social impairment were non-significant (all *p* > 0.1). Bootstrap analysis (**Table 4B**) of the specific indirect effect of focusing attention on the relationship between SA and social impairment revealed a bias corrected 95% confidence interval excluding zero (CI_0.95_ = 0.10, 0.36). Confidence intervals for the specific indirect effects of divided attention and shifting attention both included zero, indicating that the relationship between SA and social impairment is partially mediated by one specific aspect of attentional control – focusing attention. Pairwise contrasts revealed the specific indirect effect through focusing attention is larger in magnitude than the specific indirect effect through divided attention. All other pairwise contrasts were non-significant (**Table 4B**).

**Table 4B:** Mediation of the effect of social anhedonia on social impairment through the three specific components of attentional control: focusing attention, divided attention, and shifting attention.

					BC 95% CI	
Specific indirect effect	Coefficient	Point estimate	Bias	SE	Lower	Upper
**Indirect effects**
Focusing	0.207	0.205	0.002	0.080	0.098	0.366
Divided	-0.034	-0.035	-0.002	0.034	-0.136	0.006
Shifting	0.017	0.022	0.006	0.080	-0.142	0.178
Total	0.190	0.192	0.002	0.080	0.044	0.367
**Contrasts**
Focusing vs. divided	0.241	0.240	-0.0004	0.088	0.103	0.458
Focusing vs. shifting	0.191	0.183	-0.008	0.112	-0.022	0.419
Divided vs. shifting	-0.050	-0.058	-0.008	0.096	-0.249	0.131

Our theoretical model, based on prior literature, proposes that SA leads to social impairment via attentional control deficits. However, it is possible that the opposite is true – that social impairment predicts SA via attentional control deficits. Although we cannot speak to causal directions in the absence of longitudinal data, we tested this reverse mediation model in which social impairment is hypothesized to lead to SA via attentional control. Social impairment had a direct positive effect on SA (β = 0.49, SE = 0.55, *p* < 0.001) and a direct negative effect on attentional control (β = -0.38, SE = 0.06, *p* < 0.001). However, attentional control did not have a direct effect on SA in this reverse model (β = -0.13, SE = 0.09, *p* = 0.15) and the confidence intervals for the indirect effect of attentional control on SA through social impairment included zero (CI_0.95_ = -0.01, 0.13), indicating that the model in which social impairment leads to SA via attentional control is not supported by the data.

Collectively, these results indicate that attentional control, specifically focusing attention, is one of the mechanisms through which SA leads to social impairment.

## DISCUSSION

This study examined the relationship between individual differences in SA, attentional control, and social impairment in a large community sample of healthy individuals. Two main findings emerged: first, we replicated the association between high SA and high social impairment found in previous studies (Katsanis et al., 1992; Cohen et al., 2006; Blanchard et al., 2011) providing further evidence for the presence of social impairments in socially anhedonic but otherwise healthy individuals. Additionally, our results are consistent with prior evidence demonstrating attentional control deficits in high SA and schizophrenia samples, providing further support for the proposal that impaired attentional processes are characteristic of schizophrenia liability (Erlenmeyer-Kimling and Cornblatt, 1992). Second, attentional control partially mediated the relationship between SA and social impairment, accounting for 19% of the variance. Specifically, individuals with higher SA reported lower attentional control and lower social functioning. Although both attentional deficits and social impairments have been separately noted in SA, the relationship between SA, attentional control, and social impairments in this large community sample is a novel contribution to the literature. These findings suggest attentional control is one of the mechanisms underlying aberrations in the fundamental human need for social contact. Specifically, results suggest that the ability to engage attentional control processes, particularly focusing attention in the presence of irrelevant and distracting stimuli, is a cognitive feature of SA that contributes to social impairments.

Our results have implications for understanding how SA, a dimension of schizotypy that conveys risk for developing schizophrenia-spectrum disorders and a core negative symptom of schizophrenia, relates to the characteristic social impairments of the disorder and those at risk for developing it. Here, mediation analysis demonstrated attentional control is a mediator that accounts for only 19% of the variance in social impairment. This suggests that the effect of attentional control on social impairment may operate through additional variables that reflect the multiple ways that attentional control is used in social contexts. Examination of additional variables would likely account for more variance in social impairment. This is consistent with the conceptualization of attentional control mechanisms as a domain-general system that assigns control resources to facilitate multiple higher-order processes (Lesh et al., 2011), as well as literature demonstrating neurocognitive impairments affect functioning via social cognition and metacognition in schizophrenia (Schmidt et al., 2011). Socially relevant processes such as regulation of emotional information, reward processing, and metacognition are three such higher-order processes through which attentional control could indirectly influence social functioning. We discuss each one briefly below and highlight avenues for future research examining the underlying mechanisms of social impairment in SA.

The role of attentional control in the regulation of emotional information may be particularly important for understanding the pathway between attentional control deficits and social impairment. Social interactions by nature involve affectively salient information, thus deficits in the regulation of emotional information could adversely affect response to interpersonal stressors and consequently social functioning (Tully and Niendam, 2014). Our prior research is consistent with this proposal. Impaired attentional control in schizophrenia contributes to negative affective information exerting inappropriate influence on social judgments (Hooker et al., 2011), and failure to recruit neural mechanisms of attentional control predicts maladaptive responses to interpersonal conflict in healthy (Hooker et al., 2010), schizophrenia (Tully et al., 2014), and high SA samples (Hooker et al., 2014). These findings support a model in which attentional control deficits impact social functioning – particularly response to interpersonal conflict – via impaired attentional control of emotional information.

A second pathway through which attentional control could affect social functioning could be via reward processing mechanisms. For example, impaired engagement of attentional control mechanisms to down-regulate negative affective information could be accompanied by a complimentary deficit in the up-regulation of positive affective information, which is thought to underlie the anticipatory pleasure deficit in anhedonia (Pizzagalli, 2010) and could contribute to the associated reward/motivational impairments seen in high SA (Horan et al., 2008). Preliminary evidence is consistent with this proposed role for attentional control in the management of both positive and negative affect (e.g., Vasey et al., 2013). Relatedly, anticipatory pleasure deficits could also be due to an impaired ability to generate representations of the reward value of future pleasurable activities (e.g., socializing with a friend), a process dependent on attentional control functions (Burbridge and Barch, 2007). Thus, the inability to co-opt attentional control mechanisms to facilitate the generation and use of reward representations and positive affect could result in a lack of motivation to engage in pleasurable activity (Germans and Kring, 2000) and consequently negatively impact social functioning.

Finally, there is a role for metacognition – processes involved in the capacity to think about thinking, including forming representations of one’s own mental state (self-reflection) and others’ mental states (ToM; Lysaker et al., 2010a), processes that are important for successful navigation of the social world and may be closely related to attentional control processes (Fernandez-Duque et al., 2000). Impaired metacognition is considered to be characteristic of schizophrenia (Pickup and Frith, 2001) and is observable in high-risk populations (Morrison et al., 2007; Bora and Pantelis, 2013). In schizophrenia, metacognition predicts occupational (Lysaker et al., 2010a), and social functioning (Brüne et al., 2007; Lysaker et al., 2011), and mediates the relationship between neurocognition and social functioning (Lysaker et al., 2010b). Moreover, a recent study found neural activation during ToM mediates between SA and social functioning in schizophrenia participants suggesting that, at least in patient populations, SA partially impacts social functioning through ToM (Dodell-Feder et al., 2014). This may be because difficulty understanding the minds of others causes social interactions to be stressful/unpleasant, resulting in social withdrawal and/or social rejection and consequently poor social functioning (Salvatore et al., 2008). Further research examining putative relationships between SA, ToM, and social impairment are needed.

Clearly, social functioning is complex and multiply determined; a comprehensive model of how SA impacts social functioning would need to incorporate multiple neurocognitive and social cognitive factors (Schmidt et al., 2011). Here we identified attentional control as one proximal mediator of the relationship between SA and social impairment, but there is still much variance to be explained. Future research could include measures of attentional control of emotional information, reward processing, and metacognition in order to examine additional mediators of the relationship between SA and social impairment.

Limitations must be acknowledged. The sole use of self-report assessments in the current study design could have resulted in inflated relationships due to common measurement bias, thus replication samples and follow-up studies using alternative measures are necessary. In particular, the assessment of attentional control using a self-report questionnaire, although partially validated here in a sub-sample of participants and in prior literature, is less than ideal. Future studies employing behavioral assessments of attentional control are necessary. Similarly, the ability of high SA individuals to self-report their level of SA could be confounded by accompanying deficits in self-reflection/metacognition. As discussed above, future research should include measures of metacognitive processes in order to delineate the unique contribution of SA and metacognition to social impairment. Finally, our sample – although representative of the Greater Boston Community – was comprised solely of healthy individuals, warranting caution when generalizing the results to patient populations. Parallel investigations of the relationship between SA and social impairment in patient samples are needed.

It is important to note that the current study is unable to establish causal directions between variables because of its cross-sectional design; a statistically significant mediated effect does not determine the causal direction of a relationship (Preacher and Hayes, 2004). We conducted the current study on a strong theoretical foundation based on prior research demonstrating that SA predicts social impairment (Katsanis et al., 1992; Cohen et al., 2006; Blanchard et al., 2011) and that SA can be partially characterized by attentional control deficits (Giraldez et al., 2000; Barrantes-Vidal et al., 2003), leading us to hypothesize that attentional control is an underlying characteristic of high SA that explains the relationship between SA and social impairment. It is possible that the opposite is true – that social impairment impacts SA via attentional control. However, that this reverse mediation model was not significant lends further support to our theory. Although, it is possible that once social impairments are present there exists a vicious cycle whereby social impairment exacerbates/potentiates SA through some other process (e.g., reward processing, as discussed above), which in turn potentiates social impairment and so on ad infinitum. The question of causality remains, however, and only a longitudinal study design in which attentional control and SA are tracked across the life span in relation to social functioning can truly determine causal priority of the model. The New York High Risk Project partially investigated this in relation to *physical* anhedonia and attention in a longitudinal follow-up of individuals at genetic risk for schizophrenia (Erlenmeyer-Kimling et al., 1993). Results indicated that poor attentional capabilities as a child predicted physical anhedonia as an adolescent, which in turn predicted social functioning as an adult. It is possible that a similar relationship exists between the development of attentional control, SA, and social functioning. Future research should conduct longitudinal examination of these causal pathways and potential maintenance processes.

## CONCLUSION

The current study demonstrates that the relationship between SA and social impairment is partially mediated by attentional control. This has implications for our understanding of a fundamental human desire, the need to belong, and suggests attentional control is one of the mechanisms necessary for successful social interactions. This finding also illuminates one of the mechanisms underlying the relationship between a well-established negative symptom of schizophrenia and social impairment.

## Conflict of Interest Statement

The authors declare that the research was conducted in the absence of any commercial or financial relationships that could be construed as a potential conflict of interest.
